# Association Between Family Support, Coping Strategies and Anxiety in Cancer Patients Undergoing Chemotherapy at General Hospital in Medan, North Sumatera, Indonesia

**DOI:** 10.31557/APJCP.2019.20.10.3015

**Published:** 2019

**Authors:** Dina Keumala Sari, Ratna Dewi, Wardiah Daulay

**Affiliations:** 1 *Nutrition Department, Faculty of Medicine,*; 2 *Master of Nursing Program,*; 3 *Psychiatric and Mental Health Nursing Department, Faculty of Nursing, Universitas Sumatera Utara, Medan, North Sumatera, Indonesia. *

**Keywords:** Effective, public health, strategies, oncology

## Abstract

**Objective:**

This study aims to test the association between family support and coping strategies and anxiety at Dr. Pirngadi General Hospital Medan.

**Methods:**

The study was a correlational descriptive study with a cross-sectional approach. The samples were 102 cancer patients undergoing chemotherapy, who were selected through purposive sampling technique. Data was collected using a family support questionnaire instrument developed based on the concept of the House and Friedman theory, a coping strategy questionnaire modified from the Revised Ways of Coping questionnaire by Folkman and Lazarus, and an anxiety questionnaire modified from the Hamilton Anxiety Rating Scale (HAM-A). Bivariate analysis was conducted using the Product Moment correlation coefficient to test the association between family support, coping strategies, and anxiety. Multivariate analysis was conducted using the logistic regression test to investigate dominant variables associated with coping strategies and anxiety.

**Results:**

The results of the bivariate analysis showed a significant positive association (p=0.001) and strong correlation (r=0.612) between family support and Problem Focused Coping (PFC) strategy, while there was a significant negative association (p=0.001) and moderate correlation (r=-0.462) with the Emotion Focused Coping (EFC) strategy. A significant negative association (p=0.001) and strong correlation (r=-0.646) was found between family support and anxiety. The multivariate analysis showed a dominant association (p = 0.001) between family support with PFC strategy (OR = 12.2), EFC (OR = 0.142), and anxiety (OR = 0.039).

**Conclusion:**

Based on the results, it can be concluded that there was an association between good family support and effective coping strategies and lower anxiety levels in cancer patients undergoing chemotherapy. These results can be an input for health services to increase family support for cancer patients undergoing chemotherapy in combination with effective coping strategies to decrease anxiety levels.

## Introduction

Over half of cancer patients are treated with chemotherapy, which a cancer treatment that uses chemical agents or medications for both localized and metastatic cancer. Chemotherapy is usually combined with surgery or radiotherapy. Preoperative chemotherapy is performed to reduce the size of the tumor that will be removed, while postoperative chemotherapy is performed to eliminate the remainder cancer cells (Prawirohardjo, 2010).

Chemotherapy leads to various physiological and psychological side effects. The psychological side effects that may occur are including stress, anxiety, and depression. This stress leads to coping strategies performed by individuals to prevent further psychological disorders (Karabulutlu et al., 2010).

Social support, particularly from the closest family member, will affect the effectiveness of coping strategies used by cancer patients, where the presence of support from family members generally reduces the anxiety levels and improves the quality of life in cancer patients (Duci and Tahsini, 2010). A coping strategy is stated to be effective if it leads to good adaptation and a new pattern of life, while an ineffective coping strategy can lead to physical health and psychological health problems (Nasir and Muhith, 2011).

A study by Muhamad et al., (2011) found that support from all family members, especially the spouse, plays an important role in decision making and survival strategy. Another study by Kim and Morrow (2007) showed that family support affects the anxiety levels, prevention of nausea and vomiting, and their severity in cancer patients after treatment with chemotherapy.

An interview conducted with five cancer patients undergoing chemotherapy in the chemotherapy unit at Dr. Pirngadi General Hospital Medan found that three of five patients were accompanied by their family. Furthermore, two of them told that their immediate family was helpful in terms of caring about their conditions, supporting the cost and transportation of every chemotherapy session, and encouraging them to keep motivated and attend the chemotherapy sessions routinely to improve their health conditions. These patients were enthusiastic to attend chemotherapy routinely, maintained calmness during the interview, and stated that they did not have sleep disorders. In contrast, the other two patients stated that their family members did not always accompany them to the hospital, and it was not possible to always depend on the family because their family members also had other commitments. They also mentioned that they were accustomed to attend chemotherapy alone and had grown to accept this. They appeared rather unenthusiastic during the interview and stated that they had difficulties in defecation, which was once in three days, had a low appetite, experienced abdominal pains and sleep disorders, and felt sad when alone.

## Materials and Methods

The objective of this study is to test the association between family support , coping strategies, and anxiety in cancer patients undergoing chemotherapy at Dr. Pirngadi General Hospital Medan. This study was a quantitative study using a correlational descriptive design with a cross sectional approach. The correlational design is the association of two or more variables which aims to test the correlation of those variables (Polit and Beck, 2012). The study population was all cancer patients undergoing chemotherapy with a purposive sampling technique resulting in 102 individuals.

Research data was collected directly from the respondents by using questionnaires about family support, coping strategies, and anxiety. Data analysis was including univariate analysis, bivariate analysis using Pearson Product Moment correlation coefficient, and multivariate analysis using logistic regression test.

## Results

Table 1 shows characteristics of the cancer patients undergoing chemotherapy, in which most of the subjects are pre-elderly, female, married, senior high school graduates, and most of them are unemployed. The highest prevalence of cancer in the subjects was breast cancer, while the highest prevalence chemotherapy found 1-3 times.

Table 2 shows that from the 102 cancer patients undergoing chemotherapy, 70 subjects (68.6%) had good family support, while a lack of family support was observed in 32 subjects (31.4%). Focusing in Table 2, shows that from the 102 cancer patients undergoing chemotherapy, 67 subjects (65.7%) were highly likely to use the Problem-Focused Coping (PFC) strategy, while the other 35 subjects (34.3%) were less likely to use the Problem-Focused Coping (PFC). In Table 2, shows that from 102 cancer patients undergoing chemotherapy, 71 subjects (69.6%) were less likely to use the Emotion-Focused Coping (EFC) strategy, while 31 subjects (30.4%) were highly likely to use the Emotion-Focused Coping (EFC) strategy. Table 2 also shows that from 102 cancer patients undergoing chemotherapy, 53 subjects (52.0%) experienced moderate anxiety, and only 2 subjects (2.0%) experienced severe anxiety.

Table 3 shows that there was a significant association with Problem-Focused Coping (PFC) strategy (p=0.001). The correlation coefficient (r) = 0.612 indicated a strong positive correlation, which means that subjects receiving better family support were more likely to use the Problem-Focused Coping (PFC) strategy. Similarly, a significant association was observed with Emotion-Focused Coping (EFC) strategy (p=0.001). The correlation coefficient (r) = -0.462 indicated a moderate negative correlation, which means that subjects receiving better family support were less likely to use the Emotion-Focused Coping (EFC) strategy.

Table 4 shows that there was a significant association between family support and anxiety (p < 0.001). The correlation coefficient (r) = -0,646 indicated a strong negative correlation, which means that subjects receiving better family support experienced milder anxiety.

Table 5 shows that family support is a dominant variable significantly associated with the use of Problem-Focused Coping (PFC) strategy with an Odds Ratio (OR) of 12.2, which indicated that cancer patients undergoing chemotherapy with good family support were 12.2 times more likely to use the Problem-Focused Coping (PFC) strategy (fourth stage). Family support was a dominant variable significantly associated with the use of Emotion-Focused Coping (EFC) strategy with an Odds Ratio (OR) of 0.142 (OR<1 = negative association), which indicated that cancer patients undergoing chemotherapy with good family support were 0,142 times more likely to not use the Problem-Focused Coping (PFC) strategy (fifth stage).

Family support was a dominant variable, which was significantly associated with anxiety with an Odds Ratio (OR) of 0.039 (OR<1 = negative association). It means that cancer patients undergoing chemotherapy with good family support were 0.039 times more likely to experience mild anxiety (sixth stage).

## Discussion

Association between Family Support and Coping Strategies in Cancer Patients Undergoing Chemotherapy

The bivariate analysis resulted in a value of p = 0.001 indicating a significant association between family support and Problem-Focused Coping (PFC) strategy at Dr. Pirngadi General Hospital Medan, and the correlational value of r = 0.612 indicating a strong positive correlation, which means that subjects receiving better family support were more likely to use the Problem-Focused Coping (PFC) strategy.

The bivariate analysis also resulted in a value of p = 0.001 indicating a significant association between family support and Emotion-Focused Coping (EFC) strategy at Dr. Pirngadi General Hospital Medan, and the correlational value of r = -0.462 indicating a moderate negative correlation, which means that subjects receiving better family support were less likely to use the Emotion-Focused Coping (EFC) strategy.

These findings are similar to a study by Tan (2007), which showed that there was an association between social support and coping strategies in cancer patients in which a positive correlation was found between social support and Problem-Focused Coping (PFC) strategy, and a negative correlation was found between social support and Emotion-Focused Coping (EFC) strategy. Supports from all family members, particularly the spouse, had a great effect on decision making and survival strategies in managing emotions (emotional support), providing information on their health, lifestyle, and diet, and supporting the provision of facilities (instrumental support), which were helpful for breast cancer patients undergoing therapy to increase the survival of cancer patients (Muhamad et al., 2011).

Lazarus in Potter and Perry (2009) stated that individual coping strategies are determined by the type of stress experienced by the individual, individual’s life goals, beliefs about oneself and the world, and personal resources. Individuals tend to use the Problem-Focused Coping (PFC) strategy when they believe that the demand from a situation or stressor can be changed, whereas they tend to use the Emotion-Focused Coping (EFC) strategy when they believe that only a little or no changes can be done with regards to the pressure of the situation. However, in a stressful situation, an individual generally combines problem-focused coping and emotion-focused coping strategies (Lazarus et al., 2011).

Effective coping strategies in facing a chronic disease such as cancer, particularly for those undergoing treatment such as chemotherapy, will affect the compliance of the patient in attending chemotherapy sessions routinely and the presence of physical and psychological symptoms. According to the results of this study, it can be concluded that good family support is one of the personal resources that contribute to the effectively high use of Problem-Focused Coping (PFC) strategy and effectively low use of Emotion-Focused Coping (EFC) strategy, thereby increasing compliance of cancer patients to undergo chemotherapy.

*Association between Family Support and Anxiety in Cancer Patients Undergoing Chemotherapy*

Based on the bivariate analysis test, a value of = 0.001 indicates a significant association between family support and lower levels of anxiety at Dr. Pirngadi General Hospital Medan, and the correlation coefficient (r) = -0.646 indicated a strong negative correlation, which means that subjects with better family support were more likely to experience lower anxiety levels. 

These findings are similar to a study by Lekka et al., (2014), which showed that there was a moderate negative correlation between family support and anxiety, which means that better family support was associated with lower anxiety levels in lung cancer patients. Similarly, a study by Sadeghi et al., (2015) found that there was a moderate negative correlation between social support (emotional support, instrumental support, information support) and anxiety in patients undergoing hemodialysis. A study by Guan et al., (2015) showed that social support was an important factor for cancer patients undergoing treatment to decrease the anxiety levels in order to increase their quality of life.

Based on the study results, it can be concluded that good family support for cancer patients undergoing intense, cyclic, and long chemotherapy requiring repeated admissions, along with the physiological and psychological side effects that may appear, such as moderate anxiety, will decrease the anxiety levels and increase the quality of life and survivability of cancer patients. 

**Table 1:** Characteristics of Cancer Patients (n=102)

No	Characteristics	f	(%)
1	Age (Years)		
	26-45 (Adult)	32	31.3
	46-65 (Pre-elderly)	58	56.9
	>65 (Elderly)	12	11.8
2	Sex		
	Male	39	38.2
	Female	63	61.8
3	Marital Status		
	Married	75	73.5
	Not married/Widow/Widower	27	26.5
4	Education		
	Elementary School	26	25.5
	Junior High School	22	21.5
	Senior High School	47	46.1
	University	7	6.9
5	Occupation		
	Employed	48	47.1
	Unemployed	54	52.9
6	History of Chemotherapy		
	1-3	65	63.7
	>3-6	37	36.3
7	Cancer Type		
	Breast Cancer	45	44.1
	Colorectal Cancer	27	26.5
	Ovarian Cancer	15	14.7
	Nasopharyngeal Cancer (NPC)	13	12.7
	Prostate Cancer	2	2.0
	Total	102	100

**Table 2:** Frequency Distribution of Family Support, Problem Focus Coping, Emotion-Focused Coping Strategy, and Anxiety (n=102)

Parameters	f value	Percentage (%)
Family support		
Good	70	68.6
Poor	32	31.4
Problem Focused Coping (PFC) Strategy		
High	67	65.7
Low	35	34.3
Emotion Focused Coping (EFC) Strategy		
High	31	30.4
Low	71	69.6
Anxiety		
Severe	2	2.0
Moderate	53	52.0
Mild	47	46.0

**Table 3:** Result of Pearson Correlation Test of Family Support and Coping Strategies (n=102)

	Coping Strategies			
Variable	PFC		EFC	
	r	p	r	p
Family Support	0.612	0.001	-0.462	0.001

**Table 4:** Result of Pearson Correlation Test of Family Support with Anxiety (n=102)

	Anxiety	
Variable	r	p
Family Support	-0.646	0.001

**Table 5:** Result of the Stage of Logistic Regression Multivariate Analysis

Variable	Stage of LRMA*	p	Exp (B)	95% CI for Exp (B)	
				Lower	Upper
Family Support	Fourth	0.001	12.161	3.385	43.688
	Fifth	0.001	0.142	0.055	0.362
	Sixth	0.001	0.039	0.012	0.128

*LRMA, Logistic Regression Multivariate Analysis

*Dominant Variables Associated with Coping Strategies in Cancer Patients Undergoing Chemotherapy*

The multivariate analysis showed that only family support was significantly associated with Problem-Focused Coping (PFC) strategy with OR = 12.2 indicating that cancer patients undergoing chemotherapy with good family support were 12.2 times more likely to use the Problem-Focused Coping (PFC) strategy. The family support variable had a significant negative association with the Emotion-Focused Coping (EFC) strategy with OR = 0.142 indicating that cancer patients undergoing chemotherapy with lack of family support were 0.086 times more likely to use the Emotion-Focused Coping (EFC) strategy. 

These results are similar to the findings by Kim et al., (2010), which showed that social support in breast cancer patients was associated with the use of coping strategies. Lazarus et al., (2011) stated that an effective coping strategy helps an individual to tolerate and accept a psychologically stressful situation and ignore the stressors that cannot be managed.

From these results, it can be concluded that good family support for a cancer patient undergoing intense, cyclic, and long chemotherapy requiring repeated admissions will increase the compliance of the cancer patient to attend the chemotherapy to increase the quality of life.

*Dominant Variables Associated with Anxiety in Cancer Patients Undergoing Chemotherapy*

The multivariate analysis showed that only family support had a significant negative association with anxiety with an OR = 0.039 indicating that cancer patients undergoing chemotherapy with the lack of family support had the chance of 0.039 times more likely to experience severe anxiety.

This result corresponded to a study by Aberha et al., (2016), which found that the lack of social support in patients with hypertension was the most associated factor with anxiety. Social interaction plays a role in the adaptation of a patient towards chronic disease. An individual with anxiety will show poor physical, cognitive, and emotional response and is at risk of maladaptive behavior that will prevent the individual from learning various methods to alleviate anxiety. Therefore, social support, particularly from the family, may influence the individual to form new adaptive behaviors to help him/her to adapt and learn (Videbeck, 2008).

According to these results, it can be concluded that good family support received by a cancer patient undergoing intense, cyclic, and long chemotherapy with repeated admissions will help the patient to adapt and decrease their anxiety towards chemotherapy to increase their quality of life.

In conclusion, based on the study results, it can be concluded that family support was significantly associated to coping strategies (Problem-Focused Coping/PFC and Emotion-Focused Coping/EFC) and anxiety in cancer patients undergoing chemotherapy. Based on the correlation coefficient (r), there was a strong positive correlation between family support and the use of Problem-Focused Coping (PFC) strategy. Family support had a moderate negative correlation with the use of Emotion-Focused Coping (EFC) strategy. Family support had a strong negative correlation with anxiety. The better the family support, the higher the use of Problem-Focused Coping (PFC) strategy, the lower the use of Emotion-Focused Coping (EFC) strategy, and the lower the anxiety levels of the subjects. 

Multivariate analysis showed that family support in cancer patients undergoing chemotherapy became a major factor associated with the Problem-Focused Coping (PFC) strategy (OR = 12.2), which means that cancer patients with good family support were 12.2 times more likely to use the Problem-Focused Coping (PFC) strategy. However, the results for the Emotion-Focused Coping (EFC) strategy (OR = 0.142) indicated that cancer patients with good family support were 0.142 times more likely to use the Emotion-Focused Coping (EFC) strategy, and the results for anxiety levels (OR = 0.039) indicated that patients with good family support were 0.039 times less likely to experience lower levels of anxiety.

*Suggestions*

*1) For Healthcare Providers*

The study results are hoped to be used as an input for providing health education towards family members of patients on the importance of family support for cancer patients undergoing chemotherapy as part of effective use of coping strategies that will decrease the anxiety levels and increase their quality of life.

*2) For Nursing Education*

The study results can be used as additional information about the use of coping strategies (Problem-Focused Coping/PFC and Emotion-Focused Coping/EFC) and anxiety levels in cancer patients undergoing chemotherapy.

*3) For Future Researchers*

Future studies should be conducted using qualitative methods to explore family support, coping strategies, and anxiety in cancer patients undergoing chemotherapy, so that it may complement the results of existing studies.
